# Common complication of crush injury, but a rare compartment syndrome

**DOI:** 10.4103/0974-2700.62124

**Published:** 2010

**Authors:** Nissar Shaikh

**Affiliations:** Department of Anesthesia/ICU and Pain Management, Hamad Medical Corporation, Doha-Qatar

**Keywords:** Compartment syndrome, crush injury, gluteal region, neurovascular bundle, fasciotomy

## Abstract

Compartment syndrome (CS) is a common complication of crush injury but it is rare to find bilateral gluteal compartment syndrome (BGCS). Only six cases of BGCS have been reported in the literature. This syndrome has been reported after crush injury, drug overdose, surgical positioning, and vascular surgery. Apart from CS, crush injury is associated with multi-system adverse effects and these patients are at high risk for renal failure and sepsis. CS patients may present with dehydration; coagulation disorders; elevated creatine phosphokinase and myoglobin levels; hyperkalemia and hypocalcaemia, which may cause life-threatening arrhythmias and therefore need urgent and aggressive therapy. The early goal in these patients is prevention of acute renal failure with aggressive fluid therapy, alkalinization of urine, and forced diuresis. Early treatment of hyperkalemia, antibiotic therapy, immunoprophylaxis, and wound care will minimize the risk of arrhythmias and sepsis. CS must be considered when any patient is diagnosed with crush injury syndrome. CS is defined as elevation of interstitial/ intracompartmental pressure, leading to microvascular and myoneural dysfunction and secondary hypoxia; it may cause functional loss or even death if not detected early and treated properly. The increase in pressure in one or all compartments of the gluteal region causes CS with devastating effects on muscle and neurovascular bundles. CS is traditionally diagnosed on the basis of five ‘p's: pain, pallor, paraesthesia, pulselessness and paralysis. Diagnosis of gluteal CS is difficult as the peripheral pulses are preserved and the condition is usually only diagnosed when neurological abnormality is noticed. Diagnosis of CS can be made by direct measurement of the compartment pressure and magnetic resonance imaging or computerized tomography. Gluteal CS is managed by fasciotomy and debridement of necrosed tissue, with secondary closure of fascia. A high index of suspicion is necessary for the early diagnosis of gluteal CS, and this will reduce the disability and complications as a consequence of this syndrome. The acute-care physician, the intensivist, and the trauma surgeon must be aware of this rare syndrome, as it can result in multiorgan dysfunction and death. Here we report a case of bilateral gluteal CS that was successfully treated in our trauma intensive care unit.

## INTRODUCTION

The gluteal region is unique in that it contains a huge muscle mass. It is divided into three separate non-distensible compartments: the gluteus maximus compartment, the gluteus medius/minimus compartment, and the tensor fascia lata compartment.[1] Pressure may increase in one or all three compartments and lead to the compartment syndrome (CS). Increase in pressure could be due to various reasons, for example, trauma, rhabdomyolysis, drug intoxication, malpositioning during surgery, or following vascular surgery. CS is a common complication of crush injury but it is rare to find bilateral gluteal CS. Gluteal CS is a rare entity and bilateral gluteal CS is extremely rare indeed. The diagnosis of gluteal CS is difficult as peripheral pulses are preserved and the condition is usually only diagnosed when neurological abnormality is noticed. Hence, for early diagnosis of the gluteal CS, there must be a good knowledge about this condition and a high index of suspicion. Early diagnosis will reduce the disability and complications occurring as a consequence of this syndrome. It is essential for the acute care physician, the intensivist, and the trauma surgeon to be aware of bilateral gluteal CS as it has been associated with multiorgan dysfunction and even death.

Apart from causing CS, crush injuries have multisystem adverse effects and patients are at high risk for renal failure and sepsis. These patients will present with dehydration; coagulation disorders; high creatine phosphokinase (CPK) and myoglobin levels; and hyperkalemia and hypocalcaemia, which may cause life-threatening arrhythmia. These patients therefore need urgent and aggressive therapy.

Here we report a case of bilateral gluteal CS following a crush injury, which was initially thought to be a closed muscular contusion and crush injury. The patient was successfully treated in our trauma intensive care unit.

## CASE REPORT

**Figure 1:**
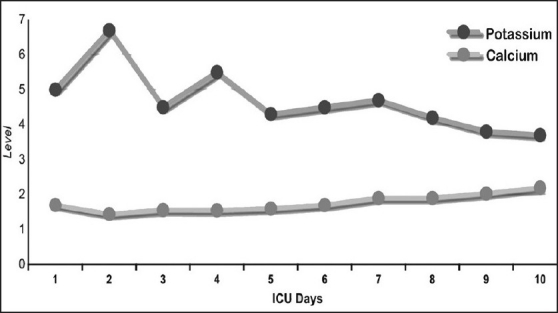
Serum potassium and calcium level in our patient

**Figure 2:**
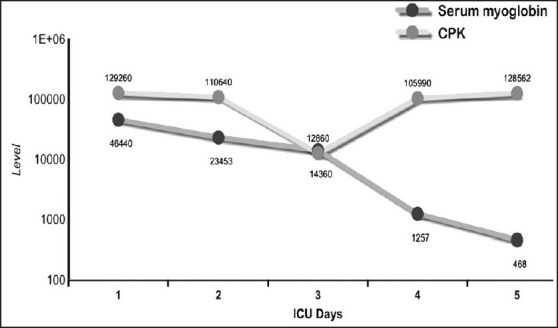
Serum myoglobin and CPK level in our patient

**Figure 3:**
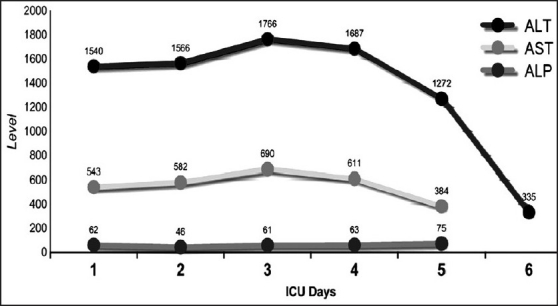
Liver functions in our patient

A 40-year-old male construction worker was trapped under a collapsed building for 7 h. After rescue, he was brought to our hospital. He was fully conscious but dehydrated and complained of severe pain in both lower limbs and buttocks. He was tachycardic but blood pressure was stable. There were multiple abrasions over both lower limbs and he had difficulty in moving them due to pain. He was passing dark-colored urine. Computerized tomography (CT) of spine, pelvis, abdomen and the skeletal survey of the lower limbs and pelvis was done but no abnormality was detected. The patient had acidosis, hyperkalemia, hypocalcaemia [Figure 1], raised serum myoglobin and CPK levels [Figure 2], and deranged liver function [Figure 3]. He was diagnosed as crush injury syndrome and admitted to the trauma intensive care unit.

Aggressive fluid therapy was started and correction of the coagulation disorder was done with blood products. Dextrose-actrapid infusions were given for the treatment of the electrolyte abnormality. Pain was controlled with intravenous tramadol. Hydration was guided by central venous pressure (CVP) monitoring. Frusemide infusion was added for forced diuresis.

On the second day of admission, his heart rate was better; coagulation became normal; muscle power at wrist, elbow, and shoulder was normal; both calf muscles were soft, but both thighs were swollen; there was no localized crepitus or collection; and all peripheral pluses were present.

On day 3, the patient complained of weakness of both lower limbs. Sensation was markedly decreased below the knee on the right side. The detailed motor examination of both lower limbs revealed the findings shown in Table 1.

**Table 1:** Neurological findings in our patient

Lower limb		Right (muscle power)	Left (muscle power)

Hip	Extensor	Grade 2	Grade 2
	Flexor	Grade 2	Grade 2
Knee	Extensor	Grade 2	Grade 2
	Flexor	Grade 2	Grade 2
Ankle	Extensor	Grade 0	Grade 2
	Flexor	Grade 0	Grade 2

The patient's electrolytes and coagulation parameters were normalized. Liver enzymes were markedly elevated and creatinine kinase (CK) was more than 100,000. Forced diuresis, sodium bicarbonate, and aggressive fluid therapy were continued. CPK and myoglobin levels started to decrease. He improved, but weakness in the lower limbs persisted. Normal diet was started and he was transferred to the orthopedic ward on day 6.

He was given adequate analgesia and was taking a normal diet but the weakness and pain in both lower limbs persisted and he was unable to walk. Electromyography (EMG) on day 16 showed bilateral sciatic nerve injury. He had fever (40°C) on day 18, and there was a greenish discharge from the abrasions over the right buttocks and thigh. Microbiology cultures from the abrasion showed pseudomonas and *Bacteroides fragilis* and, accordingly, the antibiotic was changed to meropenem. He remained febrile. Both buttocks were very tense, with edema extending to the lateral aspect of the thigh. He complained of increasing pain. Magnetic resonance imaging (MRI) on day 30 revealed bilateral multiple gluteal CS, intramuscular hematomas, and swelling of both thighs, with edema of both sciatic nerves. As MRI gave the clear diagnostic picture, we did not measure the compartment pressure, especially because measuring the multiple compartment pressures would have been cumbersome.

Patient was immediately taken to the operation room for fasciotomy of the gluteal region and right thigh and drainage of collection. He underwent debridement of necrotic tissue and muscle four times. Following this, he became afebrile. Daily dressing of the wound and physiotherapy was continued. Closure of the fasciotomy was done on day 62 and patient was started on Augmentin^®^ (amoxicillin + clavulanic acid) as per the results of the culture and sensitivity tests. He improved. The left lower limb movement became normal and the right lower limb muscle power improved, though foot drop persisted. He started to walk with support. Meropenem was discontinued on day 66. Patient was discharged home on day 70. He is being followed in the orthopedic outpatient clinic and is receiving physiotherapy for the right-sided foot drop.

## DISCUSSION

CS is a known complication of rhabdomyolysis and crush injuries, but gluteal CS is uncommon and bilateral gluteal CS is very rare. Rhabdomyolysis is common after crush injuries, which are becoming increasingly common as a result of accidents and natural calamities. Bywater and Beall[2] have given detailed descriptions of crush injuries seen during the London blitz; they have described the complications, particularly the swollen extremities, dark-colored urine, and renal failure. We will discuss the life-threatening effects and the treatment of crush injury in following text.

### Prevention of renal failure

Crush injury can cause multiorgan dysfunction, but the most commonly affected is the renal system. Crush injury commonly occurs due to trapping and compression of a part of the body, with injury—both macroscopic and microscopic—to the skeletal muscle, bleeding into the muscular compartment, edema and necrosis of the muscle. These factors lead to compromised perfusion of the muscle compartment. After resuscitation, when reperfusion occurs, myoglobin enters the circulation. Renal injury occurs mainly due to two mechanisms: first, myoglobin causes obstruction of the renal tubules and, second, the nephrotoxicity of myoglobin leads to renal vasoconstriction. Acute renal failure results if this is not treated.

To prevent the occurrence of renal failure in patients with crush injury, the early goal-directed therapy is aggressive fluid resuscitation, alkalinization of urine, and forced diuresis. Initial fluid administration should be sufficient to produce a urine output of 2 ml/kg of body weight/h. Addition of sodium bicarbonate to the resuscitation fluids is beneficial as it will correct the metabolic acidosis hyperkalemia and also keeps the urine alkaline (urinary pH > 6.5), thus decreasing the toxic effect of myoglobin on renal tubules. Acetazolamide, frusemide, and mannitol are commonly used medications for inducing forced diuresis. Frusemide has an added advantage in that it causes renal vasodilatation and decreases the oxygen demand; however, it causes renal tubular acidosis. Acetazolamide is useful when systemic alkalosis occurs, as it promotes excretion of bicarbonate ion and thus provides a double advantage. Mannitol, apart from causing an osmotic diuresis, helps in scavenging the free radicals as well as fluid from the muscular compartment.[3] The aim of forced diuresis is to maintain a urine output of 200-300 ml/kg/h by aggressive fluid administration while, at the same time, ensuring that pulmonary overload does not occur.[3]

### Measurement of compartment pressure

The gluteal CS can occur in an individual compartment or it may involve all three compartments of the gluteal region.[1] CS must be considered in any patient diagnosed with the crush injury syndrome. CS is defined as elevation of interstitial/ intracompartmental pressure, with microvascular and myoneural dysfunction and secondary hypoxia; it may cause functional loss of the affected part or even death if it is not detected early and treated properly.[4]

In the literature, only 18 cases of gluteal CS have been reported, and only six cases of bilateral gluteal CS have been published.[5] Out of these six cases, two occurred after the patient had been placed in the dorsal lithotomy position for surgery, two cases occurred after heroin-induced coma and, more recently, one case was reported after vascular surgery and another following ‘ecstasy’-induced hyperthermia.[6] Patients with crush injury have two types of injuries. First, there is direct contusion causing disruption of the cell and leading to instant death of the cell. The second type of injury is due to ischemia, which takes some time to develop; the muscle fiber becomes necrotic in 4–6 h and Wallerian degeneration occurs after approximately 8 h.[7]

**Figure 4:**
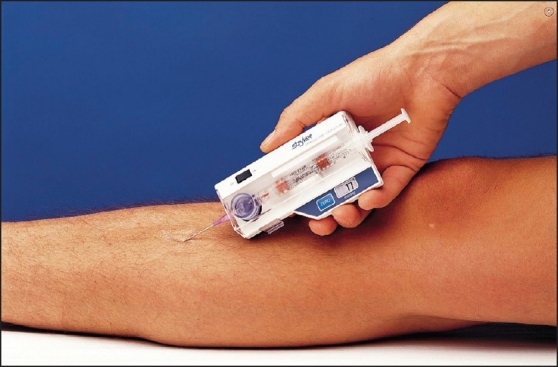
Stryker pressure monitor

Local capillary leak allows protein and fluid leakage after resuscitation, leading to tissue edema and increase in pressure within the closed compartment. This subsequently causes muscular edema, ischemia, necrosis, and compression of the neurovascular bundles, particularly of the large sciatic nerve. Neurons are particularly sensitive to pressure and hypoxia, and the first sensory deficit can occur as early as 33 min after the start of compartmental hypertension.[7] CS is traditionally diagnosed on the basis of five ‘P's. *Pain* out of proportion to the injury or precipitated by passive movement of the extremity or fingers; a *pale* and swollen affected extremity; *paresthesia* of the extremity, with the first sensation to be lost being the sense of vibration;[8] and *pulselessness* and *paralysis* of the extremity, which occurs later in the disease process. Gluteal CS is difficult to diagnose, as peripheral pulses will be present despite the increase in the compartment pressure and, besides, the other injuries that are present tend to divert the attention of the treating physician. The laboratory parameters which will be helpful in the diagnosis of CS are serum CPK and myoglobin levels. The CPK level in CS will be > 10,000 U/l. In addition, if the patient has a crush injury, there will be hyperkalemia, hypocalcaemia, acidosis, abnormal coagulation, and elevated liver enzymes.[6] The diagnosis of CS can be confirmed by measuring the compartmental pressure. One study showed that measurement of compartment pressure had a significant role in the diagnosis of CS in unconscious patients or in those unable to cooperate.[8] This pressure can be measured using commercially available instruments [Figure 4]. Alternatively, a needle can be inserted into the compartment and by connecting it to a monitor through a transducer, the pressure in that particular compartment can be measured. Uliasz *et al.* compared commercially available Stryker, Whiteside, and manometeric IV pumps in a compartment model; they found that the Stryker and the manometeric IV pump methods were accurate as standard method is, needle and transducer assembly for measuring the compartment pressure.[9] The advantage of measuring the compartment pressure is that it allows one to calculate the difference between diastolic pressure and the compartmental pressure. If this difference reaches 30 mm of Hg, fasciotomy and decompression is indicated.[10] More recently, noninvasive radiological imaging with MRI/CT has been used to accurately diagnose CS, particularly when the compartment contains bulky muscle mass, as is the case in the gluteal and thigh area.[11]

### Management of hyperkalemia

Important aspects of the management of the crush injury patient is the correction of any coagulation abnormality using blood products, treatment of hyperkalemia and hypocalcemia, and correction of acidosis. Sometimes these patients develop life-threatening arrhythmias, which should be treated promptly. Following a crush injury, hyperkalemia occurs due to massive destruction of myocytes, with massive amounts of potassium, free radicals, and lactic acid being released into the circulation. Potassium has a profound effect on the conduction system of the heart and, in combination with acidosis, it increases the risk of ventricular arrhythmias and even cardiac arrest. Hence, in patients with crush injuries, we should aggressively correct the hyperkalemia. The treatment of hyperkalemia is by administration of calcium chloride or gluconate, dextrose plus insulin infusion, sodium bicarbonate, or beta-agonist nebulization.[12]

### Management of wounds

In crush injuries, the wounds are usually contaminated with foreign bodies or necrotic debris. The extremity may be so badly crushed that it may need field amputation; alternatively, it may develop CS, in which case immediate fasciotomy is called for which, again, increases the risk of infection. To minimize the risk of infection, we should wash the wound thoroughly, examine it frequently, change the dressing repeatedly and, if necessary, carry out frequent debridement.[13]

Reis *et al*.[14] recommended that in cases of crush injuries, fasciotomy should be done whenever there is necrosis of muscle or compromised circulation. Otherwise, conservative management is preferred, as covering skin is the best protection against infection and fasciotomy will change a closed injury into an open one. In our case, as there was muscle necrosis, a ‘question-mark’ fasciotomy was done; repeated debridement was necessary and the fasciotomy was closed secondarily.

Hyperbaric oxygen therapy has been proven to be useful in crush injury patients. It improves wound healing and reduces the need for multiple surgical procedures in these patients. It delivers high-concentration oxygen to the hypoperfused tissue and thus improves tissue oxygenation.[15]

### Antibiotic of choice

Crush injury patients lose one of the main defensive barriers against infection—the skin. As a result, these wound may get contaminated with foreign body or bacteria, or the patient's own endogenous microorganisms may enter the wound. Even fasciotomy causes a loss of this natural protection against microbes.[16] If these crush injury patients goes into renal failure the risk for infection increases even further and the mortality will be high.[17]

Most commonly, crush injury wounds are infected by gram- positive streptococci or staphylococci. Kazancioglu *et al*. in their study series found that 95% of their crush injury patients developed infection with *acinetobacter* (36%) and pseudomonas species.[13] In their series they used cefazolin for clean wounds and cefazolin plus metronidazole or ampicillin/sulbactum for unhealthy looking wounds. In general, it is reasonable to start a broad-spectrum antibiotic initially and change it later according to the culture and sensitivity results. If the length of hospital stay of these patient increases due renal failure or other complications, it is important to keep in mind the possibility of nosocomial infections and to adjust the antibiotic therapy accordingly. Despite recent advances in intensive care therapy of these patients, infections are still a major worry and remained a significant factor for mortality. It is essential to take appropriate microbiological samples before starting prophylactic antibiotic therapy and adjust medication as soon as the culture and sensitivity results are available. Apart from the correct choice of antibiotics, it is also important to start the antibiotic early, usually within 30-60 min of the injury.

### Tetanus immunoprophylaxis

Tetanus is a life-threatening medical emergency but it is preventable. It is caused by *Clostridium tetani*, which is a gram-positive bacillus commonly found in soil, human feces, and on objects lying on the ground. *C tetani* enters the open wound with debris, initially causing local manifestations and then systemic manifestations. The incubation period is 2-50 days. Despite recent advances in intensive care therapy, the mortality in tetanus remains as high as 50%. Tetanus is easily preventable with the use of the highly effective vaccine.[18]

Crush injury patients are at high risk for the development of tetanus. These wounds are unhealthy as they often contain debris and necrotic material and, moreover, there is compromised perfusion of the area. All crush injury patients should be evaluated for tetanus immunoprophylaxis status. Those patients who have not been vaccinated in last 5 years should be vaccinated by either active or passive immunization. Tetanus immunoglobulin is used for passive immunization. Active immunization is done with tetanus toxoid; it is given to those patients who are not aware or sure of their immunoprophylaxis status, to immigrant population, and to elderly immunocompromised patients. Henderson *et al.*, found that none of the 11 patients in their study population who had tetanus received any recent tetanus immunization.[19]

## CONCLUSION

Aggressive fluid resuscitation, alkalinization of urine, and forced diuresis will prevent acute renal failure in crush injury patients. Early treatment of hyperkalemia, antibiotic therapy, immunoprophylaxis, and proper wound care will minimize the risk of arrhythmias and sepsis. Awareness regarding gluteal CS and a high index of suspicion is essential for the early diagnosis and effective management of these patients.
